# Snus Use in Adolescents: A Threat to Oral Health

**DOI:** 10.3390/jcm13144235

**Published:** 2024-07-19

**Authors:** Orsolya Németh, Levente Sipos, Péter Mátrai, Noémi Szathmári-Mészáros, Dóra Iványi, Fanni Simon, Márton Kivovics, Dorottya Pénzes, Eitan Mijiritsky

**Affiliations:** 1 Department of Community Dentistry, Semmelweis University, 1088 Budapest, Hungarykivovics.marton@semmelweis.hu (M.K.); penzes.dorottya@semmelweis.hu (D.P.); 2 Centre for Translational Medicine, Semmelweis University, 1088 Budapest, Hungary; 3 Doctoral School of Health Sciences, University of Pécs, 7624 Pécs, Hungary; 4 Institute for Translational Medicine, Medical School, University of Pécs, 7624 Pécs, Hungary; 5 Department of Head and Neck Surgery and Maxillofacial Surgery, Tel-Aviv Sourasky Medical Center, School of Medicine, Tel-Aviv University, Tel Aviv 64239, Israel; 6 Goldschleger School of Dental Medicine, Faculty of Medicine, Tel-Aviv University, Tel Aviv 39040, Israel

**Keywords:** snus, gingivitis, prevention, adolescents, smokeless tobacco, oral health

## Abstract

**Background:** Snus consumption is increasingly popular, mainly among the youth, due to the promotion of the tobacco industry and the lack of knowledge regarding its adverse effects. Even though some of its systemic complications are common knowledge, the oral consequences are rarely known. **Aim:** Therefore, the objective of this study is to evaluate the oral health effects of snus consumption among a highly exposed group of adolescent athletes. **Design:** Participants received an interactive presentation, followed by interviews and dental screenings by young doctors to establish trust. They were categorized into groups based on the frequency of snus usage. The oral hygiene habits and status, snus consumption habits, and awareness about its adverse effects were evaluated. **Results:** Statistically significantly (*p* < 0.05), more regular snus users experience gum bleeding while tooth brushing than nonusers (60% and 37%, *p* = 0.004). Snus consumption and poor oral hygiene have a cumulative effect on oral health. Some young athletes experience ulcerous oral mucosal lesions coinciding with snus placement. Nonusers exhibit greater awareness of the adverse effects of snus than regular users (27% and 49%). **Conclusions:** Regular snus use negatively affects oral health, especially the gums. Early education is of the utmost importance in preventing snus usage by raising awareness.

## 1. Introduction

Lately, the tobacco industry has been actively engaged in the development and promotion of novel products that could be viewed as potentially less harmful to health compared to traditional cigarettes [1]. These alternative options to smoking are gaining exceptionally high popularity among adolescents.

Snus is a moist, smokeless oral tobacco product from Sweden, where it is widely used. Usually, it is placed behind the upper lip for a while, varying from a few minutes to even more than an hour, typically many times a day [2,3]. Nicotine is absorbed from the smokeless tobacco through the oral mucosa, and the harmful components dissolving in saliva are absorbed from the intestinal walls, reaching circulation [4]. Loose and portioned sachets are available forms of snus.

In recent years, the tobacco industry has introduced and promoted novel products as potentially safer alternatives to traditional cigarettes, gaining popularity, especially among adolescents. One such product is snus, a moist, smokeless oral tobacco widely used in Sweden, placed behind the upper lip for extended periods throughout the day. Unlike most European Union countries where snus is banned, Sweden is an exception, and it is also legal in non-EU countries like Norway.

Distinct from traditional snus are Oral Nicotine Pouches (ONPs), which contain food-grade fillers, sweeteners, and flavorings, with nicotine derived from plants or synthesized, varying widely in content. Marketed as safer due to a reduced carcinogenic potential, ONPs still pose risks such as nicotine addiction and cardiovascular issues. They are marketed extensively to youth, raising concerns about increased nicotine addiction in this demographic.

The categorization of Oral Nicotine Pouches (ONPs) and traditional snus separately is crucial for understanding their distinct compositions and health implications. ONPs typically consist of food-grade fillers, sweeteners, and flavorings, with plant fibers as the primary ingredient by volume, often sourced from eucalyptus and pine. Unlike traditional snus, ONPs do not contain tobacco leaves, dust, or stems. The nicotine in ONPs can either be derived from tobacco plants or synthesized, with nicotine content varying widely across brands, typically ranging from 1 mg/pouch to 10 mg/pouch and sometimes higher [5,6].

Both ONPs and traditional snus share addictive properties, impacting oral health significantly. Our study focuses on young athletes, particularly ice hockey and football players, who are highly exposed to snus. Despite its prohibition in many places, snus remains popular among adolescents, including various synthetic alternatives. Our aim is to assess snus’s oral health consequences among this vulnerable group and emphasize the importance of prevention.

While ONPs are marketed as safer alternatives to traditional tobacco products due to their reduced carcinogenic potential, they are not without risks. Nicotine, a highly addictive substance, remains a primary concern. Users of ONPs may experience cardiovascular issues, gum damage, and nausea. Nicotine’s addictive nature can lead to dependency and contribute to cardiovascular problems such as an elevated heart rate and blood pressure. The direct exposure of oral tissues to nicotine and other chemicals in ONPs can also cause gum damage, potentially resulting in gingival recession and other oral health complications [7].

Moreover, ONPs are aggressively marketed toward younger demographics with appealing flavors and discreet use methods, often portraying them as risk-free alternatives to traditional tobacco. This targeted marketing raises concerns about increasing nicotine addiction among young users. Despite ONPs not containing tobacco, their addictive potential and associated health risks, including oral health impacts from additives, are significant concerns.

While cigarette smoking’s harmful effects are well-documented, snus’s oral health impacts are less discussed, despite associations with oral lesions, gingival recession, and other oral diseases. Prevention is crucial in mitigating these effects, especially among youth, highlighting the need for continued research and education on the risks associated with snus use [8,9,10].

While ONPs and snus differ in carcinogenic potential due to ONPs lacking tobacco, both share addictive properties and raise significant health concerns, especially among younger users. Our study focuses on young ice hockey and football players highly exposed to snus, reflecting potentially higher usage rates compared to the general population. However, our objective was not to compare ONP and snus effects but to emphasize the importance of snus prevention in any form [11].

Even though the harmful effects of cigarette smoking are generally known, the negative effects of snus, especially the oral effects, are rarely mentioned. Moreover, snus usage is sometimes even encouraged as a less harmful alternative to smoking despite the fact that it also has a carcinogenic effect and is associated with several health complications and numerous oral symptoms [12,13].

As the snus gets in contact with the oral mucosa for a longer time, usually in the exact same spot, the oral cavity is the most affected organ impaired by the side effects of snus. The utilization of the snus significantly elevates the likelihood of developing mucosal lesions [14,15]. It has also been described that smokeless tobacco use is associated with oral potentially malignant lesions (e.g., leukoplakia and erythroplakia) and different types of oral carcinomas [16]. In addition, some other oral consequences could be gingival recession, plaque accumulation, and gingivitis, leading to periodontitis [15,17,18]. Also, it is associated with a higher incidence of caries and abrasion, it causes discoloration of teeth, and regular consumers tend to have more tooth loss; however, results are still contradictory [19,20]. However, with primer prevention, the irreversible changes of the aforementioned oral diseases can be avoided.

Moreover, the available literature is very contradictory and deficient compared to smoking, and without long follow-up studies, we cannot consider it as a safe choice over smoking.

Hence, the aim of this study is to assess the oral consequences of snus (as a smokeless tobacco) use among young athletes who have significant exposure to snus. Our goal was to raise awareness and educate the participating adolescents, as all prevention methods are most beneficial in childhood.

## 2. Material and Methods

### 2.1. Participants

The sample of this study comprised adolescent Hungarian athletes, specifically ice hockey and football players involved in the national team program of their respective federations and a football academy in a major city. The adolescents were interviewed, and their health status was screened during their summer preparation sports camps. Both the participants and their legal guardians provided consent to participate in the study. This study adhered to the recommendations of the STROBE (Strengthening the Reporting of Observational Studies in Epidemiology) guidelines [21].

### 2.2. The Inclusion Criteria for the Study

In the first year, all male players, irrespective of age, who participated in the official summer training camp of the Hungarian national ice hockey team were included. In the second year, all players, regardless of gender and age, who participated in the official summer training camp of the Hungarian national ice hockey team were included. Additionally, all students of the participating football academy, irrespective of gender and age, who attended the academy’s one-week residential summer training camp during the respective years of the study were included.

### 2.3. The Exclusion Criteria for the Study

In the first year, any male players who did not participate in the official summer training camp of the Hungarian national ice hockey team and all female players were excluded. In the second year, any players who did not participate in the official summer training camp of the Hungarian national ice hockey team were excluded. For the football academy participants, any students who did not attend the academy’s one-week residential summer training camp during the respective years of the study and any underage participants without the consent of a legal guardian were excluded.

### 2.4. Setting and Study Design

This observational study consisted of three distinct phases.

The first phase of the study involved delivering an interactive presentation led by a relatable presenter to build trust and engagement. The presentation covered snus’s definition, legal aspects, effects on sports performance and oral health, addiction, and cessation strategies. Visual aids and interactive games were used to help participants remember the information. In the second phase, an 83-question survey was administered, which covered socio-demographic status, oral hygiene habits, various addictions (smoking, alcohol, drugs), factors influencing snus use, current snus habits, and awareness of its adverse effects. We used these questions to assess the short-term effectiveness of our educational program. Participants were grouped based on their snus use: regular users, occasional users, and non-users. Only regular users completed an optional part of the questionnaire about their snus habits. We also categorized participants by their oral hygiene habits (brushing twice a day or less).

The final phase was conducted by dentists who made oral screenings using disposable dental instruments. They examined each tooth and recorded data on the number of teeth, gum bleeding, fluorosis, erosion, and any soft tissue lesions. We recorded the number of decayed, missing, and filled teeth according to WHO standards. Each can range from 0 to 28. The measurements of gum bleeding were assessed by self-reporting during tooth brushing. For detection of fluorosis, we used the Dean’s Index, which ranges from 0 (normal) to 4 (severe). Erosion was evaluated using the Basic Erosive Wear Examination (BEWE), with scores from 0 (no erosion) to 3 (severe erosion affecting more than 50% of the tooth surface). Soft tissue lesions were detected and recorded during the oral screening, noting the presence, location, and type of lesions. This comprehensive data collection ensured that we accurately assessed dental health and related factors.

Participants were informed of any pathological findings to help them get care, especially for oral mucosal lesions. This approach ensured we collected comprehensive data on the effectiveness of our preventive education, the impact of snus use on health, and the risk factors for addiction. It aligns with our primary goals of identifying trends in a vulnerable group and developing prevention programs (Table 1).

This methodology ensured comprehensive data collection on the effectiveness of preventive education, the impact of snus use on health, and the identification of addiction risk factors. The approach aligns with the study’s primary objectives of identifying trends in a vulnerable population and guiding the development of prevention programs.

### 2.5. Reliability of Examiners

We evaluated the reliability of our dental examiners through inter-examiner reliability assessments. Before the study began, our dentists went through extensive calibration sessions. We used Cohen’s kappa statistics to assess inter-examiner agreement, and we achieved a value of 0.82, which indicates almost perfect agreement.

Cohen’s Kappa Index Ranges:

0.01–0.20: Slight agreement.

0.21–0.40: Fair agreement.

0.41–0.60: Moderate agreement.

0.61–0.80: Substantial agreement.

0.81–1.00: Almost perfect agreement.

To minimize bias, we also implemented standardized examination protocols and randomized participants among different examiners. Additionally, we conducted a pilot study to refine our examination procedures and address any inconsistencies

### 2.6. Statistics

The statistical evaluation was conducted using the R software version 4.1.0 summary package [22]. R is a language and environment for statistical computing and graphics. It is a GNU project similar to the S language and environment developed at Bell Laboratories (formerly AT&T, now Lucent Technologies) by John Chambers and colleagues. R provides a wide variety of statistical (linear and nonlinear modeling, classical statistical tests, time-series analysis, classification, clustering) and graphical techniques and is highly extensible. The S language is often the vehicle of choice for research in statistical methodology, and R provides an Open Source route to participation in that activity [23,24].

## 3. Results

### 3.1. Demographics

A total of 248 (12–20 years) ice hockey players and footballers participated in our study. A total of 206 male and 42 female Hungarian students attended, mostly living in a city or capital city (78%). In the self-certificate questionnaire, 20 (8.1%) of the participants considered themselves regular snus users, 36 (15%) admitted that they consume snus occasionally, and 192 (77%) stated that they have never tried snus. Only three responders admitted regular use of any other tobacco products, and three permitted occasional use of cannabis (Supplementary Table S1).

### 3.2. Knowledge about Snus

**Figure 1:**
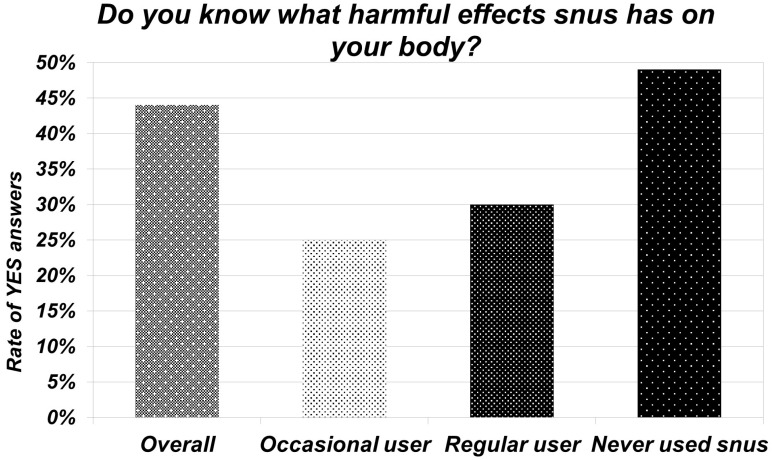
Knowledge about snus.

The questionnaire also aimed to test the adolescents’ knowledge about the negative effects of snus. The participants should answer this question in their own words. A total of 19 (44%) could specify some of the negative effects. The most widely known was the carcinogenic effect. Besides the defined oral impacts (gum and enamel-damaging effect, oral hygiene deterioration, mucosal effect, tooth loss), systemic and addictive effects were also listed. Of those who have used snus, only 15 (27%) knew about its harmful effects. In contrast, half of the group who admitted they had never tried snus were aware of its negative effects (Figure 1, Supplementary Figure S1 and Table S2).

### 3.3. Snus Habits

The vast majority of regular snus users became addicted between the ages of 14 and 16 and were regular users for 1 or 2 years. However, two claimed they were regular users since age 12, and 1 had been a snus user for six years. Furthermore, 9 (45%) used snus multiple times a day.

Portion snus was the preferred type among regular users. Most of the participants—9 (45%)—kept, on average, the snus in their mouth for 10–30 min. However, the duration of use can vary from a few minutes to more than half an hour. A total of 6 (30%) stated they usually keep multiple portions in their mouth at once (Supplementary Table S3).

### 3.4. Oral Hygiene and Dental Status of Participants

A total of 40 (15.8%) teenagers brushed their teeth only once or fewer times daily. Only 33 (14.2%) stated they used floss or interdental brush to clean the interdental areas. A total of 121 (48.2%) used only electric or manual toothbrushes or both. Other participants used other tools, namely, mouthwash or toothpicks, besides the previously mentioned interdental brushes or floss. A total of 91 (37%) patients reported that they experienced gum bleeding regularly when brushing their teeth. Even so, only five (2%) patients know about some kind of gum disease.

By the dental examination, 118 participants were diagnosed with caries. Only 80 (32%) had healthy teeth. A total of 62 patients recalled that they experienced toothache in the previous half year. The dental problems the responders experienced were a white coating on the tongue, sore gums, a bad taste, a sharp burning sensation, and a dry mouth. Some of the examined oral mucosal lesions were aphthous lesions, decubitus, and signs of morsicatio, most occurring at the buccal vestibule.

Most responders (88%) did not use any mouthguards. Those who used them used thermoplastic ones, and only a few used mouthguards made by specialists (Supplementary Table S4).

### 3.5. Oral Effects of Snus

The participants were asked if they experienced gum bleeding. The highest amount of gum bleeding was measured in the regular snus user group (60%), while in the never-used group, it was 37%, and the difference was statistically significant (*p* = 0.040) (Table 2).

The combined effect of oral hygiene habits and snus usage was also evaluated.

**Figure 2:**
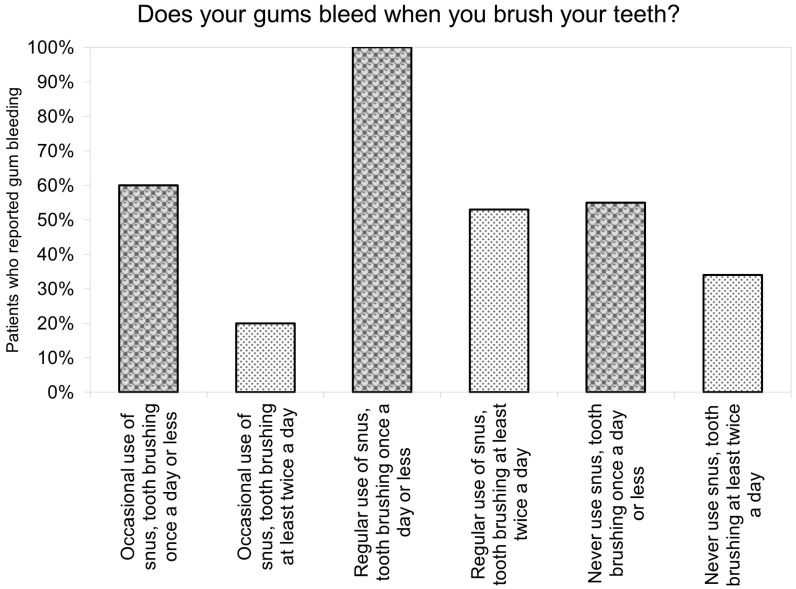
Gum bleeding associated with snus usage and oral hygiene habits.

A total of 100% of those who admitted regular snus usage and brushing their teeth only once or less reported they experienced gum bleeding. Of those regular snus users who washed their teeth more appropriately, only 53% recalled gum bleeding. This value is very similar to the value measured in the group who never used snus although had inappropriate oral hygiene. If we compare only adolescents with proper oral hygiene, those who were regular users in these groups experienced gum bleeding the most compared to the other two groups. Those who reported gum bleeding the least from all six groups were those who had never used snus and brushed their teeth twice or more (Figure 2 and Supplementary Table S5).

**Figure 3:**
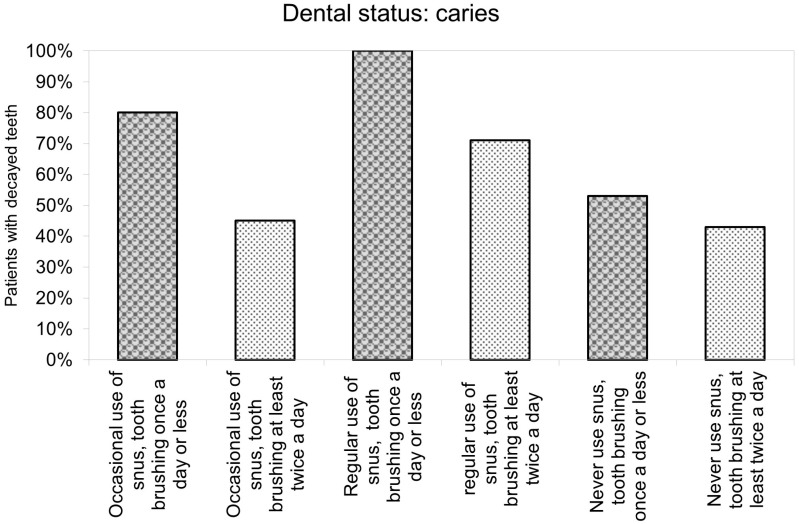
Dental caries associated with snus usage and oral hygiene habits.

The sequence of the number of patients with untreated caries is similar to those mentioned above. A total of 100% of regular snus users who had inappropriate oral hygiene had carious lesions. In those groups, where participants were either regular users or washed their teeth only once or less, more than half of the adolescents had teeth with caries. The lowest rate of carious teeth was measured in the group who had never used snus and brushed their teeth twice or more (Figure 3 and Supplementary Tables S6 and S7).

Regular snus users were asked if they had experienced ulcerative lesions in their mouth, and 30% reported that they had. A total of five reported that the location of the ulcerative lesion coincides with the site where the mucosa comes into contact with the snus regularly, and one reported a lesion elsewhere. They could also supplement the questionnaire with their own words if they have experienced other symptoms associated with snus. A total of 30% stated they had, and the specified symptoms were a sharp burning sensation in the mouth, a sore in the gum, a coated tongue, and a bad taste in the mouth.

## 4. Discussion

### 4.1. Summary of Main Findings

This study explores the correlation between snus consumption and oral health among adolescent athletes, focusing specifically on gum bleeding and caries. Our findings highlight a statistically significant difference in gum bleeding during toothbrushing among groups categorized by their snus consumption habits.

This study underscores the cumulative impact of inadequate oral hygiene and regular snus use, which is implicated in heightened gum bleeding. However, due to the limited number of participants in distinct snus user groups, our observations primarily reveal trends rather than conclusive findings.

Regarding oral health implications, while a poorer cariological status is associated with snus usage, statistically insignificant results still indicate clinically relevant differences. The prevalence of gingival bleeding observed in our cohort mirrors global trends among adolescents, emphasizing the critical role of preventive dental care in mitigating complications related to gingivitis and caries. Awareness of the detrimental effects of snus is pivotal, especially given that non-users generally exhibit higher awareness than regular users [3,6,18].

It is important to note that while this study identifies a statistically significant association between snus use and gum bleeding, causation cannot be inferred. Other factors, such as oral hygiene practices, dietary habits, systemic diseases, and additional modifying factors, could influence our results. Furthermore, being an observational study, our findings provide insights primarily applicable to our specific, highly exposed cohort of adolescent athletes.

While our study may not be fully representative of broader populations, the associations observed underscore the need for targeted interventions and continued research into the oral health impacts of snus use.

### 4.2. Importance of Prevention in Periodontitis and Oral Cancer

Gum bleeding, as a primary symptom of gingivitis, is an international challenge that impacts millions of individuals, including adolescents [25,26]. Inflammation, edema, redness, and bleeding are the characteristics of gingivitis, which is caused by the accumulation of bacterial biofilm as a result of inadequate oral hygiene [27,28]. From the health care worker aspect of professional oral hygiene treatment and from the patient side, toothbrushing and interdental brushing are important for effective prevention [29]. It is essential to provide early intervention and education on appropriate oral hygiene in order to prevent the progression of gingivitis to periodontitis [30]. Periodontitis, characterized by irreversible attachment loss, is the main cause of tooth loss in adults worldwide. Therefore, primary prevention of gingivitis through effective oral hygiene practices is essential to prevent the onset and progression of periodontal disease [31,32,33,34].

In our study, 37% of participants experienced gum bleeding, which aligns with the prevalence rates reported in other adolescent groups worldwide. It was also reported that only 15.8% of participants brushed their teeth once or less daily, while 14.2% used floss or interdental brushes, and 48.2% used either electric or manual toothbrushes. Analyzing the outcomes of our research in relation to the relevant literature, we can observe the consistency of the trends found: in the field of gingivitis and dental caries, the risk factors detected, and the preventive measures recommended, our research is in line with international findings. The studies by Olczak-Kowalczyk et al. (2019) and Fan et al. (2021) highlighted significant variations in oral health status and hygiene practices among adolescents from different regions and lifestyles, providing a useful comparison to our own study. Olczak-Kowalczyk et al. focused on 15-year-old Polish adolescents, reporting a 29.6% prevalence of gingival bleeding and signs of inflammation and gingivitis. In contrast, Fan et al.’s study of 1502 adolescents revealed a higher prevalence of gingivitis at 37.4%, correlating with infrequent tooth brushing and a lack of interdental cleaning. The identified risk factors for gingivitis varied but consistently included poor oral hygiene; plaque accumulation; and lifestyle factors such as smoking, poor diet, and stress. Fan et al. also highlighted the influence of socioeconomic status, dietary habits, and regional differences, whereas our study emphasized the combined effect of oral hygiene habits and snus usage. All studies suggest targeted preventive measures, including better education on oral hygiene, increased access to dental care services, and public health interventions to address both socioeconomic disparities and specific risk factors such as snus use [35,36] (Table 3).

Our study investigated the oral mucosa changes associated with regular snus use among respondents. A significant finding was that 30% of regular snus users reported experiencing ulcerative lesions in their mouths, with the majority indicating these lesions occurred at sites where snus came into direct contact with oral mucosa. This finding raises concerns about possible links to oral cancer, such as recurring ulcerous lesions and leukoplakia, which are considered precancerous lesions [37,38]. Oral cancer may occur in different parts of the oral cavity, such as the surface of the tongue, palate, inside of the cheeks, gingiva, or lips [39].

Numerous studies have investigated the association between regular snus use and oral cancers; however, there is still an unmet need for more studies to establish a conclusive link between snus use and oral mucosal lesions and oral cancer. This reflects the ongoing debate in the scientific community about the health effects of smokeless tobacco products [40,41,42,43]. Nevertheless, more studies could establish a link between oral mucosal lesions and snus usage [15,44]. These mucosal changes are also preventable if the triggering cause (snus) is omitted [44]. Though not all types of oral cancer can be eliminated by avoiding carcinogenic habits, most are preventable. The etiologic factors for squamous cell carcinoma, which accounts for approximately 90% of all oral malignancies, are tobacco intake (including both smoking and smokeless tobacco products) and alcohol consumption [39]. Therefore, primary prevention, most likely before adulthood, is the key to avoiding oral cancers.

Our study investigated the oral health effects associated with regular snus use among respondents. A significant finding was that 30% of regular snus users reported experiencing ulcerative lesions in their mouths, with the majority indicating that these lesions occurred at sites where snus came into direct contact with oral mucosa. This aligns with previous reviews, which consistently show a strong association between current snuff use and oral mucosal lesions, particularly in Scandinavian populations where the prevalence reaches nearly 100%. Comparing our findings with other studies, the prevalence of ulcerative lesions among snus users varies widely. Notably, the prevalence of these lesions varies by region, with Scandinavian users showing higher rates compared to those in the USA, likely due to differences in product types and usage patterns [44].

The localized nature of the lesions reported in our study, predominantly at the site of snus placement, underscores the importance of considering product placement and frequency of use in assessing oral health risks associated with snus. This finding is consistent with the notion that direct contact of tobacco products with oral tissues can lead to mucosal irritation and subsequent ulceration [45].

Furthermore, our study revealed that additional symptoms reported by snus users included a sharp burning sensation in the mouth, sore gums, a coated tongue, and a persistent bad taste. These symptoms are consistent with known side effects of snus use documented in the literature [46].

Most of the regular users in this sample have been users for 1 or 2 years; however, some participants have been users for 4 or 6 years. Miluna et al. confirmed in their study that there is a statistically significant correlation between how many years tobacco products were used and mucosal changes that could be seen in patients using tobacco products for 5–10 years [47]. It is essential to educate teenagers at an early age about the negative effects of snus, as the duration of regular usage is crucial in developing irreversible changes.

In summary, we can conclude that the battle against addictions—drugs, alcohol, and different types of tobacco products—can be the most successful with primary prevention methods in childhood. Therefore, most desirably, the manifestation of irreversible consequences can be avoided. Our study contributes to the growing body of evidence linking snus use with adverse oral health outcomes, including ulcerative lesions and associated symptoms. These findings underscore the need for public health initiatives aimed at raising awareness about the potential oral health risks associated with snus use and promoting cessation strategies among users.

### 4.3. Efficacy of Education

Prevention is a complex mission; one of its keystones is raising awareness through education. Our main goal was to educate the adolescents with an entertaining presentation, maintain their attention, and pass on as much information as possible. Even though the questionnaire was administered after the presentation, 56% of the participants admitted they did not know any negative effects of snus use. Efforts to raise awareness among adolescents about snus-related health risks reveal gaps in knowledge, indicating a need for more effective educational strategies. It is often a huge challenge to preoccupy teenagers with a presentation, as they quickly get bored with lots of information. The potential incorporation of gamification in presentations is proposed to enhance engagement and information retention. It was shown that students tend to participate more in lectures that are gamified, become more proactive, and find the material easier to learn than conventional courses [44].

Additionally, the promotion of snus is very harmful, as young people tend to believe advertisements, and the regular use of snus might become a gateway to cigarette addiction. Therefore, with the opposite effect, it will not be the planned “healthy alternative of cigarettes” [45,46].

### 4.4. Special Population Characteristics

Our sample’s unique composition adds value to our findings. The sample contains athletes, ice hockey players, and footballers, who are more exposed to snus. Some camps take place in Sweden, and their role models could be Swedish ice hockey players, where snus usage is widespread and legal. Consequently, the prevalence of regular snus use is highly above the European estimated average (1.1%), even above the Swedish general population average (12.3%) [47,48,49]. However, caution is advised in interpreting prevalence rates due to the potential under-reporting influenced by legal restrictions, as snus trade is illegal in European Union members, except Sweden [5]. The tobacco-free nicotine pouches, as substitutes for snus products, are widely advertised and sold or can be purchased online easily—their effect is considered identical in this article, but the legal environment is different for these products. Nevertheless, it should be mentioned that it can be suspected that in our examined sample, in reality, even more teenagers use snus than those who admitted due to the fear of punishment.

### 4.5. Strengths and Limitations

Regarding the strengths of our analysis, we highlight the population selection: this study focuses on a specific population—adolescent athletes—who are highly exposed to snus, providing a unique perspective on the impact of snus on oral health in this particular group. It is a cohort where diseases are still preventable due to the young ages of participants. The preventive measures included a comprehensive lecture that educated participants on the risks associated with snus and smoking, emphasizing strategies to maintain oral health, thus transcending the scope of a typical observational study.

Reliability: The robust sample size of nearly 250 adolescents enhances the credibility of our findings. Moreover, the involvement of young resident doctors and dental students in administering the questionnaire not only facilitated data collection but also fostered trust and rapport with the participants. The interactive nature of the presentation further engaged the adolescents, ensuring active participation and enhancing the overall reliability of the study.

Clear presentation of findings: our study successfully identified a statistically significant association between snus usage and gum bleeding. Additionally, we examined the combined impact of inadequate oral hygiene practices and snus use, which collectively exacerbate gum bleeding and contribute to the caries index.

Considering the limitations of this work, we observed a limited representation of regular snus users, which restricted our ability to draw definitive conclusions about this subgroup. Consequently, our analysis primarily focused on identifying trends within this subgroup’s results.

Additionally, the categorization of snus use frequency groups relied on self-assessment by participants, which may have introduced overlap between the regular and occasional user groups. For example, some adolescents classified themselves as occasional snus users based on social context but later revealed more frequent usage patterns, blurring the distinction between occasional and regular use categories.

Challenges in differentiating and simplifying oral hygiene categories also posed limitations. Factors such as varying toothbrushing frequencies and techniques among participants complicated the categorization of oral hygiene habits. Our study simplified this assessment primarily based on toothbrushing frequency, potentially overlooking nuances in oral hygiene practices that could influence oral health outcomes.

## 5. Conclusions

To summarize, snus use has a detrimental impact on oral health and is statistically significantly associated with gum bleeding. When inadequate oral hygiene practices are combined with snus use, it further exacerbates the adverse effects on oral health, particularly affecting the gums. Moreover, it is essential to recognize that oral health is closely linked to overall health and well-being. Consequently, achieving a comprehensive state of systemic health becomes challenging without proper oral health. Increasing awareness at an early age plays a vital role in reducing snus usage, and education serves as a primary means of prevention.

## 6. Bullet Points

1. Snus use negatively affects oral health and is statistically significantly associated with gum bleeding.
2. Insufficient oral hygiene and snus use cumulatively affect oral health, especially the gums.
3. Awareness contributes to reducing snus usage; therefore, early childhood education has the leading role in prevention.

## Figures and Tables

**Table 1:** Three distinct phases of observational study.

	Interactive Preventive Education	Questionnaire Administration	Individual Oral Screening
Topic	interactive presentation to build trust	an 83-question survey administration	oral screenings with dental instruments
Content	snus definition, legal aspects, effects on sports addiction, cessation strategies	socio-demographic status, oral hygiene habits, addictions, factors influencing snus use, current snus habits, adverse effects	data on teeth, gum bleeding, fluorosis, erosion, soft tissue lesions
Exercises	visual aids, interactive games	assessing the short-term effectiveness	informations about any pathological findings
Groups		grouped based on their snus use (regular users, occasional users, and non-users)	
		categorized participants by their oral hygiene habits	

**Table 2:** Gum bleeding associated with snus usage frequency.

	Overall	Occasional User	Regular User	Never Used Snus	*p*-Value
Do your gums tend to bleed when brushing your teeth?	244 (98%)				0.040
Yes	91 (37%)	9 (26%)	12 (60%)	70 (37%)	
No	153 (63%)	26 (74%)	8 (40%)	119 (63%)	
NA	4	1	0	3	

**Table 3:** Comparison of relevant literature and present study.

Study	Olczak-Kowalczyk et al. [35]	Fan et al. [36]	Németh et al.
**Sample of study**	Polish adolescents, 15 years old	Adolescents from Guangdong Province, Southern China	Ice hockey players and footballers, aged 12–20, mostly living in Hungarian urban area
**Number of participants**	Not specified	1502	248 (206 males and 42 females)
**Prevalence of gingival bleeding, signs of inflammation, and gingivitis (%)**	29.6%	37.4%	Overall: 37% Regular snus users: 60%
**Findings on oral hygiene**	Many adolescents practiced regular tooth brushing, but interdental cleaning was less common	Infrequent tooth brushing and lack of interdental cleaning correlated with increased gingivitis rates	15.8% brushed teeth once or less daily 14.2% used floss or interdental brushes. 48.2% used electric/manual toothbrushes
**Prevalence of dental caries**	A high prevalence of dental caries was reported, with a mean DMFT (Decayed, Missing, and Filled Teeth) index of 4.2	Not specified	Prevalence of dental caries: 47.58% Healthy dentition: 32%
**Risk factors of gingivitis**	Poor oral hygiene Plaque accumulation Smoking Diabetes Stress Poor diet Genetic factors	Higher prevalence was associated with poor oral hygiene practices, socioeconomic status, and dietary habits. Risk factors: age, gender, family structure, mother’s education level, oral health knowledge and attitudes, and regional differences	Regular snus user Poor oral hygiene The combined effect of oral hygiene habits and snus usage
**Suggested preventive measures**	Targeted oral health interventions Better education on oral hygiene Increased access to dental care services	Targeted public health interventions to improve oral hygiene and address socioeconomic disparities	Enhanced educational and preventive measures to address the oral health risks associated with snus use

## Data Availability

The datasets used in this study can be found in the figures, tables, and Supplementary Materials. The whole questionnaire used in our research is also available in the Supplementary Materials.

## Supplementary Materials

The following supporting information can be downloaded at: https://www.mdpi.com/article/10.3390/jcm13144235/s1, Figure S1: Specified harmful effects of snus known by the participants; Tables S1–S7.
