# Supplementary material for: Snus Use in Adolescents: A Threat to Oral Health
Source: J Clin Med. 2024 Jul 19;13(14):4235. doi: 10.3390/jcm13144235 (PMC11277688; doi:10.3390/jcm13144235)
Supplement: Supplementary file 1 [file jcm-13-04235-s001.zip › jcm-3041866-supplementary.pdf]

Supplementary materials

Supplementary Table S1 - Demographics

Characteristic	N	Overall	Usage of snus			p-value2
		N = 248 (100%)	Occasional user	Regular user	Never used snus	
			N = 36 (15%)1	N = 20 (8.1%)1	N = 192 (77%)1	
**Sex**	248 (100%)					0.088
**male**		206 (83%)	34 (94%)	18 (90%)	154 (80%)	
**female**		42 (17%)	2 (5.6%)	2 (10%)	38 (20%)	
**Age**	246 (99%)					
**12**		2 (0.8%)	0 (0%)	0 (0%)	2 (1.1%)	
**13**		26 (11%)	0 (0%)	0 (0%)	26 (14%)	
**14**		33 (13%)	1 (2.8%)	0 (0%)	32 (17%)	
**15**		52 (21%)	2 (5.6%)	2 (10%)	48 (25%)	
**16**		53 (22%)	11 (31%)	1 (5.0%)	41 (22%)	
**17**		43 (17%)	16 (44%)	5 (25%)	22 (12%)	
**18**		20 (8.1%)	2 (5.6%)	5 (25%)	13 (6.8%)	
**19**		16 (6.5%)	4 (11%)	7 (35%)	5 (2.6%)	
**20**		1 (0.4%)	0 (0%)	0 (0%)	1 (0.5%)	
**NA**		2	0	0	2	
**Location**	248 (100%)					0.009
**village**		53 (21%)	3 (8.3%)	1 (5.0%)	49 (26%)	

**capital city**		45 (18%)	9 (25%)	4 (20%)	32 (17%)	
**capital city agglomeration**		5 (2.0%)	2 (5.6%)	1 (5.0%)	2 (1.0%)	
**abroad**		6 (2.4%)	3 (8.3%)	0 (0%)	3 (1.6%)	
**city**		139 (56%)	19 (53%)	14 (70%)	106 (55%)	
**What regular sports activities do you engage in?**	248 (100%)					<0.001
**football**		141 (57%)	10 (28%)	5 (25%)	126 (66%)	
**football, individual sport**		3 (1.2%)	0 (0%)	0 (0%)	3 (1.6%)	
**ice-hockey**		103 (42%)	26 (72%)	15 (75%)	62 (32%)	
**other team sport**		1 (0.4%)	0 (0%)	0 (0%)	1 (0.5%)	
**Highest level of education of mother**	247 (100%)					
**8th elementary school grade**		4 (1.6%)	0 (0%)	0 (0%)	4 (2.1%)	
**doctoral studies**		2 (0.8%)	0 (0%)	0 (0%)	2 (1.0%)	
**university**		77 (31%)	17 (47%)	5 (25%)	55 (29%)	
**college**		53 (21%)	5 (14%)	3 (15%)	45 (24%)	
**secondary school**		49 (20%)	9 (25%)	8 (40%)	32 (17%)	
**do not know**		31 (13%)	1 (2.8%)	3 (15%)	27 (14%)	
**no educational qualifications**		1 (0.4%)	0 (0%)	0 (0%)	1 (0.5%)	
**vocational training**		30 (12%)	4 (11%)	1 (5.0%)	25 (13%)	
**NA**		1	0	0	1	
**Highest level of education of father**	248 (100%)					
**8th elementary school grade**		3 (1.2%)	0 (0%)	0 (0%)	3 (1.6%)	

**doctoral studies**		2 (0.8%)	1 (2.8%)	0 (0%)	1 (0.5%)	
**university**		63 (25%)	14 (39%)	6 (30%)	43 (22%)	
**college**		55 (22%)	6 (17%)	4 (20%)	45 (23%)	
**secondary school**		40 (16%)	7 (19%)	6 (30%)	27 (14%)	
**do not know**		31 (13%)	0 (0%)	3 (15%)	28 (15%)	
**vocational training**		54 (22%)	8 (22%)	1 (5.0%)	45 (23%)	
**Do you use any other tobacco products?**	248 (100%)					0.538
Yes		3 (1.2%)	1 (2.8%)	0 (0%)	2 (1.0%)	
No		245 (99%)	35 (97%)	20 (100%)	190 (99%)	
**Do you consume articles containing marijuana/cannabis?**	248 (100%)					0.074
Occasionally		3 (1.2%)	2 (5.6%)	0 (0%)	1 (0.5%)	
No		245 (99%)	34 (94%)	20 (100%)	191 (99%)	
**Do you consume drugs other than marijuana?**	248 (100%)					>0.999
No		247 (100%)	36 (100%)	20 (100%)	191 (99%)	
Do not want to answer		1 (0.4%)	0 (0%)	0 (0%)	1 (0.5%)	

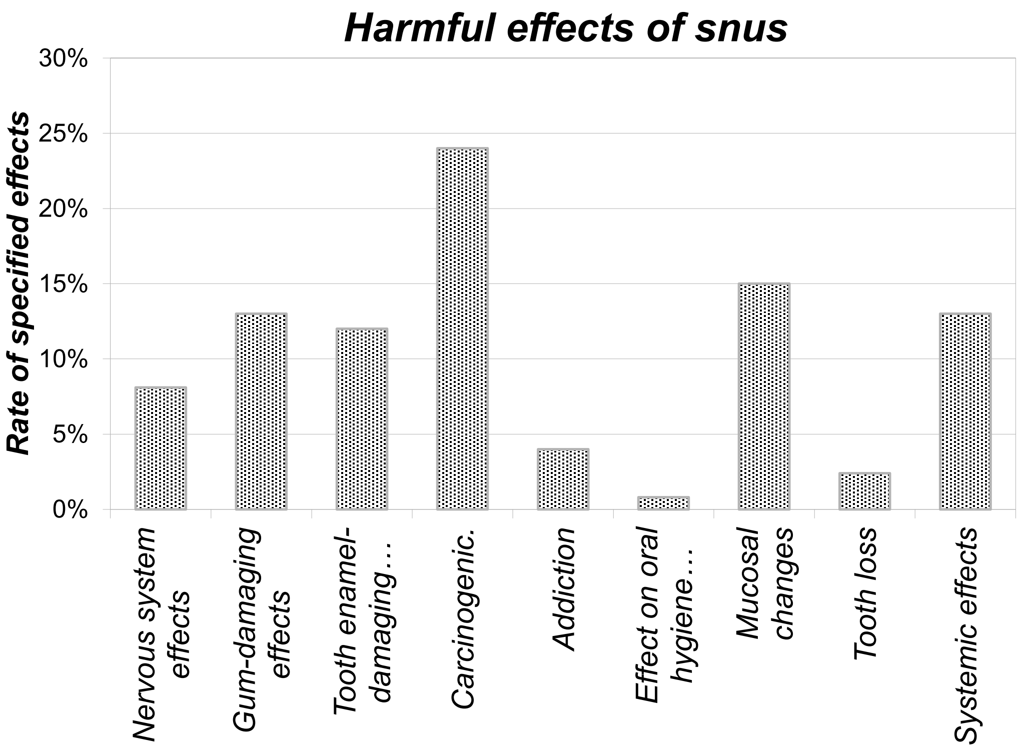

Supplementary Figure S1 - Specified harmful effects of snus known by the participants

Supplementary Table S2 - Harmful effects of snus

**Do you know what harmful effects snus has on your body? - nervous system effects**	248 (100%)					0.131
Yes		20 (8.1%)	3 (8.3%)	4 (20%)	13 (6.8%)	
No		228 (92%)	33 (92%)	16 (80%)	179 (93%)	
**Do you know what harmful effects snus has on your body? - Gum-damaging effects**	248 (100%)					0.017
Yes		32 (13%)	10 (28%)	3 (15%)	19 (9.9%)	

No		216 (87%)	26 (72%)	17 (85%)	173 (90%)	
**Do you know what harmful effects snus has on your body? - Tooth enamel-damaging effects**	248 (100%)					0.639
Yes		29 (12%)	5 (14%)	3 (15%)	21 (11%)	
No		219 (88%)	31 (86%)	17 (85%)	171 (89%)	
**Do you know what harmful effects snus has on your body? - carcinogenic.**	248 (100%)					<0.001
Yes		60 (24%)	20 (56%)	7 (35%)	33 (17%)	
No		188 (76%)	16 (44%)	13 (65%)	159 (83%)	
**Do you know what harmful effects snus has on your body? - addiction**	248 (100%)					0.014
Yes		10 (4.0%)	4 (11%)	2 (10%)	4 (2.1%)	
No		238 (96%)	32 (89%)	18 (90%)	188 (98%)	
**Do you know what harmful effects snus has on your body? - effect on oral hygiene deterioration**	248 (100%)					0.176
Yes		2 (0.8%)	0 (0%)	1 (5.0%)	1 (0.5%)	
No		246 (99%)	36 (100%)	19 (95%)	191 (99%)	
**Do you know what harmful effects snus has on your body? - mucosal changes**	248 (100%)					0.813

Yes		37 (15%)	4 (11%)	3 (15%)	30 (16%)	
No		211 (85%)	32 (89%)	17 (85%)	162 (84%)	
**Do you know what harmful effects snus has on your body? - tooth loss**	248 (100%)					>0.999
Yes		6 (2.4%)	1 (2.8%)	0 (0%)	5 (2.6%)	
No		242 (98%)	35 (97%)	20 (100%)	187 (97%)	
**Do you know what harmful effects snus has on your body? - systemic effects**	248 (100%)					0.007
Yes		31 (13%)	2 (5.6%)	7 (35%)	22 (11%)	
No		217 (88%)	34 (94%)	13 (65%)	170 (89%)	
**Do you know what harmful effects snus has on your body? - Do not know**	247 (100%)					0.011
Yes		109 (44%)	9 (25%)	6 (30%)	94 (49%)	
No		138 (56%)	27 (75%)	14 (70%)	97 (51%)	

Supplementary Table S3 – Snus consumption habits

	**N**	**Overall**	**Occasional user**	**Regular user**	**p-value[2]**
		248 (100%)	36 (15%)	20 (8.1%)	
**When did you first try snus?**	56 (23%)				0.263
12-13		2 (3.6%)	0 (0%)	2 (10%)	
13-14		4 (7.1%)	3 (8.3%)	1 (5.0%)	
14-15		18 (32%)	11 (31%)	7 (35%)	

15-16		18 (32%)	14 (39%)	4 (20%)	
16 years old or older		14 (25%)	8 (22%)	6 (30%)	
NA		192	0	0	
**What type of snus do you use?**	8 (3.2%)				>0.999
Portion/ pouch/ sachet		6 (78%)		6 (78%)	
Filtered		1 (13%)		1 (13%)	
Loose		1 (13%)		1 (13%)	
NA		240	36	12	
**Where do you place it inside your mouth?**	20 (8.1%)				>0.999
Between the upper lip and the gum		19 (95%)		19 (95%)	
Between the upper lip and the gum, between the lower lip and the gum		1 (5.0%)		1 (5.0%)	
NA		228	36	0	
**On average, how many minutes do you keep a portion of snus in your mouth?**	20 (8.1%)				>0.999
<10 minutes		7 (35%)		7 (35%)	
10-30 minutes		9 (45%)		9 (45%)	
30-60 minutes		2 (10%)		2 (10%)	
Variable		2 (10%)		2 (10%)	
NA		228	36	0	

**Do you usually keep multiple portions in your mouth at once?**	20 (8.1%)				>0.999
Yes		6 (30%)		6 (30%)	
No		14 (70%)		14 (70%)	
NA		228	36	0	
**How often do you use snus?**	20 (8.1%)				>0.999
Occasionally		6 (30%)		6 (30%)	
A few times a week		1 (5.0%)		1 (5.0%)	
Daily		4 (20%)		4 (20%)	
Multiple times a day		9 (45%)		9 (45%)	
NA		228	36	0	
**How many years have you been using it regularly?**	17 (6.9%)				>0.999
0		1 (5.9%)		1 (5.9%)	
1		7 (41%)		7 (41%)	
2		5 (29%)		5 (29%)	
3		1 (5.9%)		1 (5.9%)	
4		2 (12%)		2 (12%)	
6		1 (5.9%)		1 (5.9%)	
NA		231	36	3	

Supplementray Table S4 - Oral status, oral hygiene habits

**Do you experience ulcerative lesions in your mouth?**	20 (8.1%)					>0.999
Yes, but they occur elsewhere		1 (5.0%)	0 (NA%)	1 (5.0%)	0 (NA%)	
Yes, where the snus comes into contact		5 (25%)	0 (NA%)	5 (25%)	0 (NA%)	
No		14 (70%)	0 (NA%)	14 (70%)	0 (NA%)	
NA		228	36	0	192	
**How often do you brush your teeth?**	248 (100%)					0.176
Every two days		2 (0.8%)	0 (0%)	0 (0%)	2 (1.0%)	
Once a day		38 (15%)	5 (14%)	3 (15%)	30 (16%)	
Twice a day		178 (72%)	31 (86%)	14 (70%)	133 (69%)	
Multiple times a day		30 (12%)	0 (0%)	3 (15%)	27 (14%)	
**What tools do you use?**	248 (100%)					
Electric toothbrush		38 (15%)	9 (25%)	3 (15%)	26 (14%)	
Toothbrush + other (e.g., wooden toothpick)		19 (7.7%)	1 (2.8%)	1 (5.0%)	17 (8.9%)	
Toothbrush + dental floss / interdental cleaner		16 (6.5%)	2 (5.6%)	1 (5.0%)	13 (6.8%)	
Toothbrush + dental floss / interdental cleaner + mouthwash		17 (6.9%)	0 (0%)	0 (0%)	17 (8.9%)	
Toothbrush + mouthwash		53 (21%)	10 (28%)	3 (15%)	40 (21%)	
Toothbrush + mouthwash + other		22 (8.9%)	3 (8.3%)	4 (20%)	15 (7.8%)	

Manual + electric toothbrush		13 (5.2%)	1 (2.8%)	3 (15%)	9 (4.7%)	
Manual toothbrush		70 (28%)	10 (28%)	5 (25%)	55 (29%)	
**What toothpaste do you use?**	248 (100%)					0.279
Fluoride-free		9 (3.6%)	2 (5.6%)	2 (10%)	5 (2.6%)	
Fluoridated		92 (37%)	11 (31%)	6 (30%)	75 (39%)	
Doesn't know, but uses toothpaste		147 (59%)	23 (64%)	12 (60%)	112 (58%)	
**Do your gums tend to bleed when brushing your teeth?**	244 (98%)					0.040
Yes		91 (37%)	9 (26%)	12 (60%)	70 (37%)	
No		153 (63%)	26 (74%)	8 (40%)	119 (63%)	
NA		4	1	0	3	
**Fluorosis**	248 (100%)					0.305
healthy		92 (37%)	9 (25%)	9 (45%)	74 (39%)	
mild		1 (0.4%)	0 (0%)	1 (5.0%)	0 (0%)	
questionable		2 (0.8%)	0 (0%)	0 (0%)	2 (1.0%)	
moderate		1 (0.4%)	0 (0%)	0 (0%)	1 (0.5%)	
missing		1 (0.4%)	0 (0%)	0 (0%)	1 (0.5%)	
very mild		3 (1.2%)	1 (2.8%)	0 (0%)	2 (1.0%)	
Not examinated		148 (60%)	26 (72%)	10 (50%)	112 (58%)	
**Have you noticed any of the following symptoms? - coated tongue, white coating**	85 (34%)					0.562
Yes		29 (34%)	2 (18%)	4 (36%)	23 (37%)	
No		56 (66%)	9 (82%)	7 (64%)	40 (63%)	

NA		163	25	9	129	
**Have you noticed any of the following symptoms? - bad taste in the mouth**	85 (34%)					0.432
Yes		26 (31%)	5 (45%)	2 (18%)	19 (30%)	
No		59 (69%)	6 (55%)	9 (82%)	44 (70%)	
NA		163	25	9	129	
**Have you noticed any of the following symptoms? - sore on the gum**	85 (34%)					0.403
Yes		27 (32%)	2 (18%)	5 (45%)	20 (32%)	
No		58 (68%)	9 (82%)	6 (55%)	43 (68%)	
NA		163	25	9	129	
**Have you noticed any of the following symptoms? - sharp, burning sensation.**	85 (34%)					<0.001
Yes		8 (9.4%)	1 (9.1%)	5 (45%)	2 (3.2%)	
No		77 (91%)	10 (91%)	6 (55%)	61 (97%)	
NA		163	25	9	129	
**Have you noticed any of the following symptoms? - dry mouth**	85 (34%)					0.499
Yes		20 (24%)	1 (9.1%)	2 (18%)	17 (27%)	
No		65 (76%)	10 (91%)	9 (82%)	46 (73%)	
NA		163	25	9	129	

**How often do you visit the dentist?**	248 (100%)					
Only when it hurts		41 (17%)	5 (14%)	2 (10%)	34 (18%)	
Yearly		72 (29%)	16 (44%)	3 (15%)	53 (28%)	
Every six months		76 (31%)	12 (33%)	4 (20%)	60 (31%)	
More frequently than every six months		48 (19%)	3 (8.3%)	9 (45%)	36 (19%)	
Less frequently than yearly		11 (4.4%)	0 (0%)	2 (10%)	9 (4.7%)	
**Are you aware of gum problems?**	247 (100%)					0.002
Yes		5 (2.0%)	1 (2.8%)	3 (15%)	1 (0.5%)	
No		242 (98%)	35 (97%)	17 (85%)	190 (99%)	
NA		1	0	0	1	
**Do you use a mouthguard?**	247 (100%)					<0.001
Only during training		1 (0.4%)	1 (2.9%)	0 (0%)	0 (0%)	
only during matches		15 (6.1%)	5 (14%)	3 (15%)	7 (3.6%)	
Yes, regurarly		14 (5.7%)	5 (14%)	3 (15%)	6 (3.1%)	
Do not use		217 (88%)	24 (69%)	14 (70%)	179 (93%)	
NA		1	1	0	0	
**mouthguard/ dental splint usage**	247 (100%)					<0.001

Use		30 (12%)	11 (31%)	6 (30%)	13 (6.8%)	
do not use		217 (88%)	24 (69%)	14 (70%)	179 (93%)	
NA		1	1	0	0	
Note: The low number of patients (30, 12%) using mouthguard was due to the obligation for children to wear a cage to protect their heads from injuries. Therefore, almost no traumatic tooth loss could be noticed.						
**Mouthguard type**	248 (100%)					<0.001
Individually crafted by a professional		11 (4.4%)	1 (2.8%)	3 (15%)	7 (3.6%)	
Do not use		218 (88%)	25 (69%)	14 (70%)	179 (93%)	
Not professionally crafted		18 (7.3%)	9 (25%)	3 (15%)	6 (3.1%)	
do not know		1 (0.4%)	1 (2.8%)	0 (0%)	0 (0%)	
**Dental condition**	248 (100%)					0.183
Tooth loss		11 (4.4%)	1 (2.8%)	1 (5.0%)	9 (4.7%)	
Decay		118 (48%)	18 (50%)	15 (75%)	85 (44%)	
Completely healthy teeth		80 (32%)	13 (36%)	2 (10%)	65 (34%)	
Filling, no decay		39 (16%)	4 (11%)	2 (10%)	33 (17%)	
**Gum bleeding (groups)**	248 (100%)					
< 10%		48 (19%)	2 (5.6%)	6 (30%)	40 (21%)	
> 50%		13 (5.2%)	1 (2.8%)	4 (20%)	8 (4.2%)	
10 - 20%		21 (8.5%)	6 (17%)	1 (5.0%)	14 (7.3%)	
20 - 50%		25 (10%)	6 (17%)	2 (10%)	17 (8.9%)	
NA		141 (57%)	21 (58%)	7 (35%)	113 (59%)	

**Tooth erosion**	241 (97%)					0.275
No sign		238 (99%)	33 (100%)	19 (95%)	186 (99%)	
Enamel lesion		3 (1.2%)	0 (0%)	1 (5.0%)	2 (1.1%)	
NA		7	3	0	4	

Supplementary Table S5 – The combined effect of snus usage and oral hygiene habits on gum bleeding

		**Overall**	**Usage of snus and tooth brushing**						
		N = 248 (100%)	**Occasional use of snus, tooth brushing once a day or less**	**Occasional use of snus, tooth brushing at least twice a day**	**Regular use of snus, tooth brushing once a day or less**	**Regular use of snus, tooth brushing at least twice a day**	**Never use snus, tooth brushing once a day or less**	**Never use snus, tooth brushing at least twice a day**	
**Characteristic**	**N**		N = 5 (2.0%)*1*	N = 31 (13%)*1*	N = 3 (1.2%)*1*	N = 17 (6.9%)*1*	N = 32 (13%)*1*	N = 160 (65%)*1*	**p-value***2*
**Does your gums bleed when you brush your teeth?**	244 (98%)								0.003
yes		91 (37%)	3 (60%)	6 (20%)	3 (100%)	9 (53%)	17 (55%)	53 (34%)	
no		153 (63%)	2 (40%)	24 (80%)	0 (0%)	8 (47%)	14 (45%)	105 (66%)	

Supplementary Table S6 - Dental caries

		**Overall**	**Occasional user**	**Regular user**	**Never used snus**	**p-value**
**Dental condition**	248 (100%)					0.183
Tooth loss		11 (4.4%)	1 (2.8%)	1 (5.0%)	9 (4.7%)	
Decay		118 (48%)	18 (50%)	15 (75%)	85 (44%)	
Completely healthy teeth		80 (32%)	13 (36%)	2 (10%)	65 (34%)	
Filling, no decay		39 (16%)	4 (11%)	2 (10%)	33 (17%)	

Supplementary Table S7 - The combined effect of snus usage and oral hygiene habits on dental caries

		Overall	Occasional use of snus, tooth brushing once a day or less	Occasional use of snus, tooth brushing at least twice a day	Regular use of snus, tooth brushing once a day or less	Regular use of snus, tooth brushing at least twice a day	Never use snus, tooth brushing once a day or less	Never use snus, tooth brushing at least twice a day	p-value
**Dental status**	248 (100%)								0.529
Tooth loss		11 (4.4%)	0 (0%)	1 (3.2%)	0 (0%)	1 (5.9%)	2 (6.3%)	7 (4.4%)	
Decay		118 (48%)	4 (80%)	14 (45%)	3 (100%)	12 (71%)	17 (53%)	68 (43%)	
Completely healthy		80 (32%)	1 (20%)	12 (39%)	0 (0%)	2 (12%)	7 (22%)	58 (36%)	
Filled, not decayed		39 (16%)	0 (0%)	4 (13%)	0 (0%)	2 (12%)	6 (19%)	27 (17%)	

For all tables above:

1 n (%)
2 Fisher's exact test
* Fisher's Exact Test for Count Data with simulated p-value (based on 2000 replicates)
